# Clinico-epidemiological features of the hospitalized patients with 2009 pandemic influenza A (H1N1) virus infection in Saurashtra region, India (September, 2009 to February, 2010)

**DOI:** 10.4103/0970-2113.76294

**Published:** 2011

**Authors:** Rajesh K. Chudasama, Umed V. Patel, Pramod B. Verma, Chikitsa D. Amin, Dinkar Savaria, Rakesh Ninama, Nilesh Fichadiya

**Affiliations:** *Department of Community Medicine, Government Medical College, Rajkot, Gujarat, India*

**Keywords:** Epidemiologic information, H1N1 subtype, influenza A virus, reverse transcriptase polymerase chain reaction

## Abstract

**Background:**

The first case of 2009 pandemic influenza A (H1N1) virus infection in India was reported in May, 2009 and in Saurashtra region in August, 2009. We describe the clinico-epidemiological characteristics of patients who were hospitalized with 2009 influenza A (H1N1) infection in Saurashtra region.

**Materials and Methods:**

From September, 2009 to February, 2010, we observed 274 persons infected with 2009 influenza A (H1N1) virus who were admitted in different hospitals in Rajkot city. Real-time reverse-transcriptase-polymerase-chain-reaction (RT-PCR) testing was used to confirm infection; the clinico-epidemiological features of the disease were closely monitored.

**Results:**

Of 274 patients, median age was 29.5 years, and 51.5% were males. Only 1.1% patients had recent travel history to infected region. Median time of five days was observed from onset of illness to influenza A (H1N1) diagnosis, while median time of six days reported for hospital stay. All admitted patients received oseltamivir drug, but only 16.1% received it within two days of onset of illness. One fourth of admitted patients were expired. The most common symptoms were cough (96.7%), fever (92%), sore throat and shortness of breathing, and coexisting conditions including diabetes mellitus (9.9%), hypertension (8.8%), chronic pulmonary diseases (5.5%) and pregnancy (5.5%) (*P*<0.05). Pneumonia was reported in 93% patients with chest radiography.

**Conclusion:**

We have demonstrated that infection-related illness affects both children and adults with survival of 74% patients. The median time from onset of illness to virus detection with use of real-time RT-PCR is five days. Pregnancy is found as a significant (*P*<0.05) risk factor for severe disease.

## INTRODUCTION

Influenza A (H1N1) (earlier known as swine flu) is a new influenza virus causing illness in human beings. First detected in Mexico in April, 2009, it was originally referred as “swine flu” because many of the genes in this new virus were found in pigs in United States (US). Further on, it has been found that this new virus has gene segments from the swine, avian and human flu virus genes. The scientists call this a ‘quadruple reassortant’ virus and hence this new (novel) virus is christened “Influenza A (H1N1) virus”.[1–3] The World Health Organization (WHO) raised the pandemic level from 5 to 6, the highest level after the documentation of human to human transmission of the virus in at least three countries in two of the six world regions defined by the WHO.[4,5]

The first case of confirmed infection with the virus in India was documented in May, 2009,[6] but only few cases were reported up to August, 2009. After that large numbers of positive cases were reported throughout the India. From Gujarat state, first H1N1 positive confirmed case was reported in June 2009.[7] Saurashtra region is a western part of Gujarat state, and reported first case in August 2009.[8] All patients with confirmed infection were quarantined in isolation ward to prevent spread in the general population. This report summarizes the clinical and epidemiological characteristics of 274 confirmed cases of 2009 pandemic influenza A (H1N1) virus infection, hospitalized in various hospitals of Rajkot city of Saurashtra region from September, 2009 to February, 2010.

## MATERIALS AND METHODS

A total of 274 patients found to be positive and admitted in different hospitals of Rajkot from 1^st^ September, 2009 to 20^th^ February, 2010 were included for analysis.

### Categorization of influenza A (H1N1) cases[9]

Ministry of Health and Family Welfare, Government of India had issued guidelines for categorization of influenza A (H1N1) cases during screening for home isolation, testing treatment, and hospitalization, Table 1.

**Table 1:** Categorization of influenza A (H1N1) patients as per clinical features

Category and clinical features	Antiviral treatment	RT-PCR* testing and hospitalization
Category A		
Mild fever, cough/sore throat, body ache, headache, diarrhea, vomiting. Patient should be monitored and reassessed after 24 to 48 h	Not needed	Not needed
Category B (1)		
Signs of category A, and/or high grade fever, severe sore throat. Home isolation is advisable	May be given	Not needed
Category B (2)		
Signs of category A, and/or any of the high risk conditions like, children with mild illness but with predisposing risk factors; pregnant women; persons aged 65 years or more; patients with lung, liver, hear, kidney diseases, blood disorders, diabetes, neurological disorders, cancer, HIV/AIDS; long term steroid therapy	Given	No testing required but hospitalization may be needed
Category C		
In addition to signs and symptoms of category A and B, any of the following: breathlessness, chest pain, drowsiness, fall in blood pressure, sputum mixed with blood, bluish discoloration of nails; children with red flag signs like somnolence, high and persistent fever, inability to feed well, convulsions, shortness of breath, difficulty in breathing; worsening of underlying chronic conditions	Start immediately	Immediate testing and hospitalization

* RT-PCR: Reverse transcriptase polymerase chain reaction

In current report, total 274 patients belonging to category C were tested confirmed, hospitalized, monitored and included in the analysis.

### Clinical case /suspected case definition

A suspected case was defined as an influenza like illness (temperature≥37.5°C and at least one of the following symptoms: sore throat, cough, rhinorrhea, or nasal congestion) and either a history of travel to a country where infection had been reported in the previous seven days or an epidemiologic link to a person with confirmed or suspected infection in the previous seven days. A confirmed case was defined by a positive result of a real-time reverse transcriptase polymerase chain reaction (RT-PCR) assay performed at a laboratory operated under the auspices of the state government.[5] A close contact was defined as a person who lived with or was exposed to the respiratory secretions or other bodily fluids of patients with suspected or confirmed influenza A (H1N1) infection.

### Variables

Several types of data collected from the patients, including demographic, any coexisting conditions, regarding onset of illness and treatment, were taken. Data regarding hospitalization, whether intensive care needed, duration of antivirus drug, and disease outcome were collected from medical records and statistics departments of various hospitals.

### Data management

Data collection and analysis were coordinated by the Community Medicine Department, P D U Medical College, Rajkot. All admitted patients’ admission history and their medical records were assessed from swine flu ward for initial clinico-epidemiological details and from medical records and statistics departments after patient discharge/death from Civil Hospital and various private hospitals of Rajkot city. Line list number was given to every patient to avoid duplication at any time during study period. We made no assumptions regarding missing data; all proportions were calculated as percentages of the patients with available data. Approval by institutional review board was not required, because this infectious disease was covered under epidemic act and state health department[10] has implemented Epidemic Disease Control Act, 1897 from 18^th^ August, 2009 and issued a notification that it was in the interest of the public health to collect data on an emerging pathogen.

### Laboratory confirmation of infection

The 2009 H1N1 virus was detected with the use of a real time RT-PCR assay in accordance with the protocol from the US centers for Disease Control and Prevention, as recommended by the WHO.[11] Two swabs from naso-pharynx and one from pharynx were collected from suspected patients and their contacts for detection of influenza A (H1N1) virus by real-time RT-PCR assay.

### Statistical analysis

Continuous variables were summarized as means (±SD). For categorical variables, the percentages of patients in each category were calculated and appropriate statistical test (chi square test) was applied when required. We calculated descriptive statistics for all study variables. All data was entered in MS Excel, and analyzed by using Epi Info software (version 3.5.1) from CDC.[12]

## RESULTS

### Demographic and clinical characteristics of patients

From 1^st^ September, 2009 to 20^th^ February, 2010, a total of 274 human cases of infection with 2009 H1N1 influenza A [Table 2] were diagnosed. Positive cases were reported mainly from Rajkot city (47.8%), followed by Rajkot district (26.6%), while 70 (25.6%) cases were from other districts of Saurashtra region namely, Junagadh, Kutch, Jamnagar, Porbandar, Amreli, Surendranagar and Bhavnagar.

**Table 2:** Baseline characteristics, disease history, and outcomes of 274 patients infected with 2009 pandemic influenza A (H1N1) virus in Saurashtra region

Characteristics	Value – no. (%)
Age in year	
Median	27 yrs
Range	4.5 mths – 68 yrs
Age group of positive patients	
< 15 years	59 (21.5)
15-44 yrs	150 (54.8)
45-64 yrs	59 (21.5)
> 65 yrs	6 (2.2)
Sex	
Male	141 (51.5)
Female	133 (48.5)
Recent travel to infected region*	3 (1.1)
Hospital stays in days	
Median (in days)	6
<2 days	41 (15.0)
3-5 days	72 (26.3)
6-10 days	115 (42.0)
>11 days	46 (16.8)
Time interval from onset of illness to hospital admission and diagnosis	
Median (in days)	5
<1 day	18 (6.6)
1-4 days	115 (42.0)
5-10 days	129 (47.1)
>10 days	12 (4.4)
Antiviral treatment	
Any antiviral drug received	274 (100.0)
<2 days after onset of symptoms	44 (16.1)
Outcome of patients	
Survived	203 (74.1)
Expired	71 (25.9)
Time interval from antivirus drug started to death	
<1 day	7 (9.9)
1-4 days	35 (49.3)
5-10 days	15 (21.1)
>10 days	14(19.7)

* An infected region was defined as an area where one or more confirmed cases of 2009 pandemic influenza A (H1N1) virus infection had been found in the preceding 7 days

**Figure 1:**
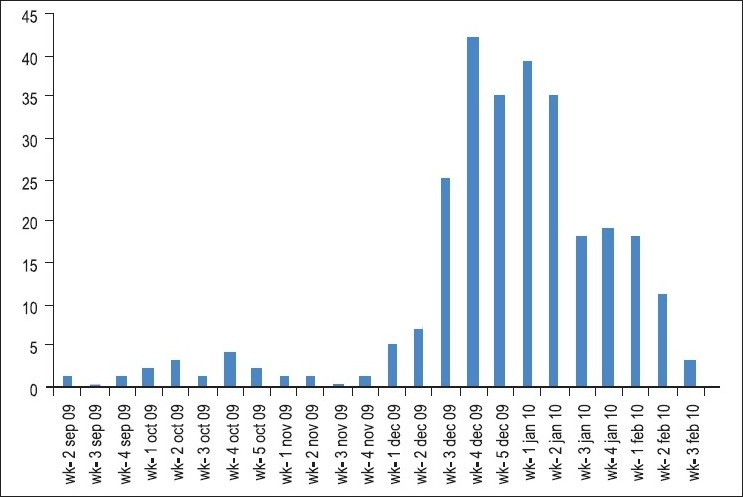
Week wise distribution of infected influenza A (H1N1) cases from September 2009 to February 2010 in Saurashtra region

Week wise distribution [Figure 1] of influenza A (H1N1) infected patients in Saurashtra region shows that number of cases increases gradually from first week of December, 2009 onwards. But from third week of December, 2009, sudden increase was seen with highest positive cases (42) reported in fourth week of December, 2009 which then remains at high level during January, 2010 followed by a gradual fall in number of positive cases in February, 2010.

The median age of 27 years was reported (range 4.5 months to 68 years) in positive cases. The median duration of diagnosis of infection was five days after onset of illness (1-20 days range). Of the 274 patients, majority of the patients reported with cough (97%) and fever (92%), followed by complaint of sore throat (54%) and shortness/difficulty in breathing (53%) [Table 3]. Totally, 91 (33.2%) cases had an underlying medical condition. Diabetes mellitus (DM) (9.9%) and hypertension (8.8%) either alone or together was mainly reported as the underlying condition, while chronic pulmonary diseases (asthma, chronic obstructive pulmonary disease (COPD), tuberculosis) were reported in 5.5% cases. Among reported female patients, 15 (5.5%) were pregnant with a range of 5 to 9 months of amenorrhea.

**Table 3:** Clinical features and coexisting conditions of 274 influenza A (H1N1) infected patients in Saurashtra region

Characteristics	Value – no. (%)
Clinical features	
Cough	265 (96.7)
Fever (≥37.5° Celsius)	252(92.0)
Sore throat	149 (54.4)
Shortness/difficulty in breathing	146 (53.3)
Nasal catarrh	68 (24.8)
Headache	59 (21.5)
Vomiting	27 (9.9)
Coexisting conditions	
Any one condition	90 (32.8)
Diabetes mellitus	27 (9.9)
Hypertension	24 (8.8)
Chronic pulmonary diseases (Asthma, COPD, Tuberculosis)	15 (5.5)
Pregnancy	15 (5.5)
Chronic heart diseases	13 (4.7)
Seizure disorder	7 (2.6)
Chronic renal failure	2 (0.7)

### Laboratory and radiographic findings

Leukopenia was observed in 24.8% of 238 patients, and lymphopenia in 66.3% of adult patients and 21.7% of children [Table 4]. Among the admitted patients, 34.6% of 240 patients reported anemia, of which 9.6% had severe anemia (Hb <8 gm/dl) and 14.6% moderate anemia (Hb – 8-9.9 gm/dl). Thrombocytopenia was found in 22.9% of 214 patients tested. Chest X-ray was done in 227 (82.8%) admitted patients and among them pneumonia was found in 93% patients.

**Table 4:** Laboratory and radiographic findings on hospital admission in influenza A (H1N1) infected patients*

Characteristic	No. /Total No. (%)
Leukocyte count	
Mean count	7335±5127
Leukopenia (<4,000/ mm^3^)	59/238 (24.8)
Leukocytosis (>10,000/ mm^3^)	51/238 (21.4)
Anemia	
Mild (10.0-11.0 gm/dl)	25/240 (10.4)
Moderate (8-10 gm/dl)	35/240 (14.6)
Severe (<8 gm/dl)	23/240 (9.6)
Lymphocyte count	
<1500/ mm^3^ in adults	118/178 (66.3)
<3000/ mm^3^ in children	13/60 (21.7)
Platelet count	
Mean count	226,000+133,000
Thrombocytopenia (<150,000/ mm^3^)	49/214 (22.9)
Thrombocytosis (>350,000/ mm^3^)	27/214 (12.6)
Elevated alanine aminotransferase (>40 U/liter)	
Any deviation	99/115 (86.1)
Elevated aspartate aminotransferase (>40 U/liter)	
Any deviation	36/110 (32.7)
Chest X-ray findings	
Done	227/274 (82.8)
Pneumonia found	211/227 (93.0)
Antibiotic treatment received	242/274 (88.3)
Corticosteroid treatment received	126/274 (46.0)

* Plus-minus values are mean ± SD

### Treatment outcome

The median time for hospital stay was six days for influenza A patients (H1N1). Duration of hospital stay of six days or more was observed in 58.8% patients. All the reported positive patients had received antiviral drug oseltamivir [Table 2]. Out of 274 positive patients, 16.1% received antiviral drug within two days of onset of illness. After hospital admission, 74% cases survived and discharged, while 26% cases were expired even after receiving treatment including antiviral drugs and life saving support. Among 71 expired patients, more than half of the patients (53.5%) were from productive age group 15-45 years. More than 40% patients were expired even after receiving complete course (five days) of antiviral drug oseltamivir.

Age fifteen years or less, presence of any coexisting condition, pregnancy, radiologically confirmed pneumonia were significantly associated (*P*<0.05) with severe influenza A (H1N1) patients who need either intensive care or died then among non severe cases, Table 5.

**Table 5:** Characteristics of non severe and severe influenza A (H1N1) hospitalized patients

Characteristics	Non severe influenza A (H1N1)* (N=187)	Severe influenza A (H1N1)† (N=87)
Age		
Median – yr (range)	27 (0.5-68)	27 (0.5-68)
<15 years – no. (%)‡	19 (10.2)	43 (49.4)
Clinical features – no. (%)		
Cough	180 (96.3)	85 (97.7)
Fever	171 (91.4)	81 (93.1)
Shortness of breath	96 (51.3)	50 (57.5)
Coexisting conditions – no. (%)		
Any one condition‡	53 (28.3)	37 (42.5)
Diabetes Mellitus	17(9.1)	10 (11.5)
Hypertension	17(9.1)	7 (8.0)
Chronic pulmonary diseases	9 (4.8)	6 (6.9)
Pregnancy‡	4 (2.1)	11 (12.6)
Seizure disorder	6 (3.2)	1 (1.1)
Pneumonia on chest radiography on admission‡ – no./total no. (%)	139/155 (89.7)	72/72 (100)
Antiviral treatment received <2 days after onset of symptoms–no. (%)	27 (14.4)	17 (19.5)
Corticosteroid treatment received– no. (%)‡	72 (38.5)	54 (62.1)

* Non severe influenza A (H1N1) – patients don’t need intensive care and survived

† Severe influenza A (H1N1) – patients need intensive care or died

‡ p<0.05 on bivariate analysis (χ^2^ or Fisher’s exact test)

## DISCUSSION

This cohort study identified 274 patients with confirmed 2009 influenza A (H1N1) belonging to category C,[9] who were hospitalized in various super specialty hospitals in Rajkot from September, 2009 to February, 2010. The median age of patients was 27 years. Significant association was found for age fifteen years or less and severe influenza A (H1N1) like other countries.[13–15] In China, majority (76.5%) of the infected patients have history of travel to infected origin countries, where one or more confirmed cases of 2009 pandemic influenza A (H1N1) virus infection had been found in the preceding seven days, while in our study, only 1.1% infected patients were reported to have history of travel to such areas. The median time of hospitalization from admission to discharge or death was six days (range <1 to 52 days). From onset of illness to diagnosis of infection and hospital admission, our study reported five days median time interval, more than in other countries,[15,16] with possible justification that patients from rural areas and small town areas were initially treated at local level by general practitioners, but with no or little improvement after initial treatment and later they were referred to higher center.

The majority of 2009 H1N1 viruses that have been tested at the CDC to date have been susceptible to two neuraminidase inhibitors, oseltamivir and zanamivir, and resistant to two adamantanes, amantadine and rimantadine.[3,17] Ministry of Health and Family Welfare, Government of India has recommended and supplied oseltamivir to the state governments for distribution in tertiary care centers and district hospitals in adequate quantity and was available in reported region also. Although the evidence of benefit from antiviral therapy is strongest when treatment is initiated within 48 h after the onset of illness, a study with oseltamivir in hospitalized patients reported reduction in mortality even after 48 h of onset of illness.[18] In present study area, all the influenza A (H1N1) infected patients received oseltamivir after hospital admission, but only 16.1% had received it within two days of onset of illness. Use of antiviral drug is beneficial, especially when initiated early, since patients who were admitted to ICU or died were less likely to have received such therapy within 48 h after the onset of symptoms,[15] while our study suggest 40% mortality even after complete course of oseltamivir therapy, possibly because of delayed referral and initiation of antiviral drug.

Week wise distribution shows that cases increases gradually from first week of December, and sudden increase was reported from third week (*n*=42) of December, which then remains at high level during January followed by gradual fall during February. The atmospheric temperature remains lowest in December and January, correlating to the increase in reported number of infected patients with influenza A (H1N1). It signifies the relationship between influenza virus and cold season, as maximum number of cases occurs during these months of winter season.[14–16]

Majority of the patients reported cough and fever similar to other countries.[1,19,20] Our study had a low prevalence (33.2%) of underlying medical conditions than in US (73%),[15] with a range of 44 to 84% as reported in other studies.[18,21,22] Diabetes mellitus (9.9%) and hypertension (8.8%) were the most common underlying conditions in the patients we studied, in contrast to asthma and COPD, as reported in US.[15,23] The 5.5% prevalence of pregnancy (*P*<0.05) in this cohort was higher than the expected prevalence in the general population (1%),[24] which was slightly low (7%) then reported in US.[15] Pregnancy as a risk factor is more strongly associated (*P*<0.05) with severe disease than non severe influenza. During periods of seasonal influenza and past pandemics, pregnant women have been at higher risk for influenza associated morbidity and mortality.[24–26] Significant number of patients with severe influenza A (H1N1) reported pneumonia (*P*<0.05) on chest radiography, received antiviral drugs and antibiotics. In the absence of accurate diagnostic methods, patients who are hospitalized with suspected influenza and lung infiltrates on chest radiography should be considered for treatment with both antibiotics and antiviral drugs.[27]

Limitations: The data was taken only from hospitalized patients. Patients belonging to category B, treated on outpatient basis and not being tested, were not included. All diagnostic testing was clinically driven, and other investigations were not obtained in a standardized fashion. Despite the use of a standardized data collection form, not all information was collected for all patients. The findings may be different during future waves, owing to the timely deployment of an effective vaccine, to viral mutation, and to resistance to antiviral drugs.

## CONCLUSION

We have demonstrated that infection-related illness affects both children including infants and adults with survival of 74.1% patients. The median time from onset of illness to virus detection with use of real-time RT-PCR is five days. Pregnancy was found as a significant (*P*<0.05) risk factor for severe disease.
